# Depth Profiling of Trace Constituents Using Secondary Ion Mass Spectrometry

**DOI:** 10.6028/jres.093.088

**Published:** 1988-06-01

**Authors:** Charles W. Magee

**Affiliations:** EVANS EAST, Inc., The Office Center, Suite 1236, 666 Plainsboro Road, Plainsboro, NJ 08536

Secondary ion mass spectrometry (SIMS) offers unique capabilities for in-depth elemental characterization of thin solid films. SIMS utilizes a beam of keV-energy ions to sputter off the outermost atomic layers of a sample. By performing mass/charge analysis of the ion population of these sputtered particles, one can determine the elemental composition of the sample surface. Since the instantaneous surface recedes into the bulk as sputtering continues, monitoring of the sputtered ions as a function of time yields in-depth concentration profiles of the detected elements. The technique is capable of detecting all elements—hydrogen through uranium—with sub part-per-million sensitivity for most elements. The technique is capable of depth resolution in the 50–100 Å range even at depths greater than 10,000 Å. SIMS, since it is a mass spectrometric technique, can also yield isotopic information, and can be made quantitative through careful instrument operation and use of standards.

Unfortunately, the raw data from a SIMS depth profile are of little use in solving problems. The depth scale of a depth profile obviously depends on the sputtering rate of the material being analyzed. However, the rates at which samples are sputtered by the primary ion beam vary widely with material and sputtering conditions (e.g., ion energy, ion species, angle of incidence) and must be measured for each set of samples being analyzed. This is easily accomplished after the analysis by measuring the depth of the sputtered crater by profilometry and equating sputtering time to sputtered depth.

Transforming a secondary ion intensity into an elemental concentration is somewhat more difficult. Because elemental sensitivities in a given matrix can vary by six orders of magnitude, and given the fact that a given element’s sensitivity can vary over several orders of magnitude depending upon the matrix in which it is found, standards are needed for virtually all elements in the matrix to be analyzed. Fortunately since SIMS is an inherently linear technique with small backgrounds, only one standard per element is required. Standards made by ion implantation are particularly good for SIMS because the number of impurity atoms added to the matrix is very accurately known (usually to within 1–2%), and virtually any element can be implanted into virtually any matrix. Using these methods, elemental concentrations in a SIMS depth profile can be measured to within 10–20% on a routine basis, and accuracies of better than 5% are possible. Depth scales on SIMS depth profiles can be measured to better than 5% given a standard of sufficient accuracy with which to calibrate the profilometer. Accuracies of this order in both concentration and depth are necessary in many semiconductor applications. Junction depths (depths into the semiconductor at which both p- and n-type dopants are of equal concentration) can be obtained from SIMS depth profiles, as well as areal densities of dopants (measured in atoms/cm^2^ calculated by integrating the area of the profile of atoms/cm^3^ vs depth in cm). Accuracies of a factor of two are not sufficient for these exacting applications.

## Applications

Determination of total areal densities of dopants in semiconductors is often needed. Whether determining dopant areal densities of diffused species for use with electrical measurements (e.g., spreading resistance), or determining total retained dose of an ion-implanted sample which has undergone subsequent thermal processing, the high sensitivity of SIMS combined with its excellent depth resolution make it the clear technique of choice for these kinds of analyses.

SIMS is also the technique of choice in certain applications requiring highly accurate, high sensitivity analyses but without the need for depth resolution (e.g., a bulk analysis). One such example is the determination of oxygen grown into highly doped silicon boules. In normal Si material, which is grown doped p- or n-type at approximately 0.1 ppma, the oxygen concentration is usually measured by FTIR using ASTM procedure F121-80. Oxygen levels are of the order of 15 ppma. However, in highly doped material used for latch-up resistant CMOS circuits, the FTIR method cannot be used, yet the oxygen content of this material must be very rigidly controlled. SIMS has been shown to be able to determine the oxygen content in highly doped wafers of this type with accuracies of 10% relative at the 15 ppma level with counting statistics of better than 1%.

Semiconductor samples serve well to illustrate the extreme sensitivity of the SIMS technique. A depth profile was taken on a sample of GaAs which had been ion implanted with Si at an energy of 70 keV to a dose of 4.5E12/cm^2^. Such a sample is quite commonly used in microwave technology. The profile of the implanted Si had a maximum concentration of only 10 ppma and was detected down in concentration to approximately 20 ppba. The truly remarkable fact about this analysis was that the data were taken from an area on the sample 200 micrometers square, and data were taken in depth increments of 20 Å. This resulted in an analyzed volume per data point of only 1E-10 cc or 1E-7 microliters in terms more familiar to organic chemists. In this extremely small volume, however, the sought-for-constituent was present at only 20 ppba, resulting in a detected amount of Si per data point of only 1E5 atoms, or 2E-19 moles, again in terms more familiar to organic chemists. There are very few other analytical techniques commonly practiced today which exhibit this kind of elemental sensitivity.

The excellent depth resolution of SIMS in combination with its sensitivity have made it indispensable in understanding the subtleties of ion implantation into semiconductors. Long ago SIMS showed that the depths of penetration of ions into silicon were not as predicted by early theoretical models. Subsequent models (e.g., SUPREM-III from Stanford University) have actually used SIMS depth profiles to refine their predictions of what the in-depth distribution of the implanted species should be. It was also shown by SIMS how an appreciable fraction of the implanted ions are guided into the channels between the rows of atoms in the single crystal samples used in the semiconductor industry. This caused the actual depth distribution of the implanted ions to be quite different from that calculated by even the newest models used to predict ion-implant profiles. SIMS also showed how this unwanted “ion channeling” effect could be eliminated by rendering amorphous by ion bombardment the near-surface region of the crystal in which the ion implant was to reside. This has recently proven very important as device engineers try to keep the implanted dopants very close to the crystal surface which is important as dimensions of transistors shrink.

## Conclusion

It is hoped that the reader has acquired some appreciation of the problems, yet power of quantitative depth profiling using secondary ion mass spectrometry. The technique needs standards to obtain the accuracy needed for most of the applications at which it has excelled, and no doubt, this is a serious problem. Yet, in the field of analytical chemistry there exist very few techniques which exhibit quantitative accuracies in the 10–20% range without the use of standards, (techniques such as atomic absorbtion certainly require standards). These problems with quantitation are more than made up for by the technique’s sub-ppm sensitivity, and universal applicability in terms of both sample type and elemental coverage, especially when one considers that this degree of sensitivity and accuracy is obtainable with depth resolution in the 50–100 Å range.

